# NMR-Based Metabolomic Analysis of Sera in Mouse Models of CVB3-Induced Viral Myocarditis and Dilated Cardiomyopathy

**DOI:** 10.3390/biom12010112

**Published:** 2022-01-11

**Authors:** Qing Kong, Jinping Gu, Ruohan Lu, Caihua Huang, Xiaomin Hu, Weifeng Wu, Donghai Lin

**Affiliations:** 1 Department of Cardiology, The First Affiliated Hospital of Guangxi Medical University, Nanning 530021, China; kongkq@126.com; 2 Key Laboratory for Chemical Biology of Fujian Province, MOE Key Laboratory of Spectrochemical Analysis & Instrumentation, College of Chemistry and Chemical Engineering, Xiamen University, Xiamen 361005, China; jingpingu@foxmail.com (J.G.); lrh626718@163.com (R.L.); xmuhuxm@163.com (X.H.); 3 Key Laboratory for Green Pharmaceutical Technologies and Related Equipment of Ministry of Education, College of Pharmaceutical Sciences, Zhejiang University of Technology, Hangzhou 310014, China; 4 Research and Communication Center of Exercise and Health, Xiamen University of Technology, Xiamen 361024, China; huangcaihua@xmut.edu.cn

**Keywords:** viral myocarditis, dilated cardiomyopathy, metabolomics, ^1^H-NMR, coxsackievirus, B3 virus

## Abstract

Viral myocarditis (VMC) is an inflammatory heart condition which can induce dilated cardiomyopathy (DCM). However, molecular mechanisms underlying the progression of VMC into DCM remain exclusive. Here, we established mouse models of VMC and DCM by infecting male BALB/c mice with Coxsackievirus B3 (CVB3), and performed NMR-based metabonomic analyses of mouse sera. The mouse models covered three pathological stages including: acute VMC (aVMC), chronic VMC (cVMC) and DCM. We recorded ^1^D ^1^H-NMR spectra on serum samples and conducted multivariate statistical analysis on the NMR data. We found that metabolic profiles of these three pathological stages were distinct from their normal controls (CON), and identified significant metabolites primarily responsible for the metabolic distinctions. We identified significantly disturbed metabolic pathways in the aVMC, cVMC and DCM stages relative to CON, including: taurine and hypotaurine metabolism; pyruvate metabolism; glycine, serine and threonine metabolism; glycerolipid metabolism. Additionally, we identified potential biomarkers for discriminating a VMC, cVMC and DCM from CON including: taurine, valine and acetate for aVMC; glycerol, valine and leucine for cVMC; citrate, glycine and isoleucine for DCM. This work lays the basis for mechanistically understanding the progression from acute VMC to DCM, and is beneficial to exploitation of potential biomarkers for prognosis and diagnosis of heart diseases.

## 1. Introduction

Viral myocarditis (VMC) is characterized by myocardial inflammation. About 10–20% of patients with VMC may progress to dilated cardiomyopathy (DCM), a frequent cause of cardiac failure and a terminal heart condition requiring transplantation [1]. As two distinct pathological stages of heart diseases, VMC and DCM cause significant health problems worldwide with an estimated incidence rate of myocarditis up to 1–2% [2,3,4].

Previous works have indicated that Coxsackievirus B3 (CVB3) acts as one of the primary pathogens of VMC [3,4]. CVB3 is a member of the family Picornaviridae and genus Enterovirus with nonenveloped, linear and positive-sense ssRNA. The CVB3 infection can trigger persistent immune responses in acute VMC (aVMC), and induce autoimmunity and chronic cardiac inflammation, thereby resulting in DCM [5,6]. As reported previously, infection of the susceptible BALB/c mice with CVB3 could well mimick human CVB3 infection, which could develop aVMC from week 1–2 post-infection, and chronic VMC (cVMC) from week 3–8 after infection, and then a dilation of the heart ventricle (DCM) several months after the CVB3 infection [7]. These mouse models have been extensively used to study the progression of VMC into DCM, and provide mechanistic understandings of pathogenic immune and inflammation responses related to VMC and DCM [7,8]. However, efficient therapeutic measures have not been developed to alleviate the progression from acute VMC to DCM due to both the unclear molecular mechanisms underlying the pathological progression and the lacks of specific diagnostic and prognostic biomarkers at an early stage [9,10].

Recently, metabolomic analyses have been emerging as useful and efficient tools for accessing metabolic processes in heart tissues, identifying potential biomarkers for diagnosis and prognosis of heart diseases, and addressing primary metabolic alterations in disease onset and progression, as well as clarifying molecular mechanisms underlying the pathogenesis of heart diseases [11,12]. For example, Halade et al. showed that lipoxygenase drives lipidomic and metabolic reprogramming with significantly changed plasma amino acids and biogenic amines in ischemic heart failure (HF) after myocardial infarction, facilitates cardiac healing and thereby improve survival [13]. Diguet et al. exhibited that the Nicotinamide riboside treatment increases myocardial levels of three metabolites (nicotinic acid adenine dinucleotide, methyl-nicotinamide, and N1-methyl-4-pyridone-5-carboxamide), which can be exploited as biomarkers for evaluating the treatment of DCM [14].

Furthermore, Müller et al. indicated that circulating metabolites, particularly fatty acids, can reflect cardiac metabolic defects which deteriorate heart functions. Increasing carnitine can significantly improve ejection fraction and reduce interstitial fibrosis in mice subjected to transverse aortic constriction (TAC) [15]. More importantly, rat models display apparent metabolic shifts in different pathological stages of HF from compensated hypertrophy stage to decompensated hypertrophy stage. Similarly, patients with HF also show obvious metabolic changes from stage A to stage B and subsequently stage C. For example, glycolysis metabolism acts as core nodes in stage A; both alanine metabolism and fatty acid metabolism are key nodes in stage B; both glucose-alanine cycle and cysteine metabolism are central connections in stage C. Significantly disturbed metabolic pathways with profoundly altered metabolite levels potentially contribute to molecular mechanisms of the HF pathogenesis, and provide potential biomarkers and therapeutic intervention targets for HF treatments [16].

Therefore, few works have been reported on metabolomic analysis for the progression of VMC into DCM. In the present work, we established the mouse models of VMC and DCM by infecting BALB/c mice with CVB3 following the procedure described in published references [7,8]. We selected mice without infection at week 2, week 6 and week 24 as normal controls (CON-w2, CON-w6, CON-w24), corresponding to these three pathological stages (aVMC, cVMC and DCM), respectively. We performed NMR-based metabonomic analyses of mouse sera, compared metabolic profiles and significantly disturbed metabolic pathways in these pathological stages relative to their metabolite levels, and identified significant metabolites and counterparts. This work may be beneficial to mechanistically understanding the progression of VMC into DCM and exploring potential biomarkers for prognosis and diagnosis of heart diseases.

## 2. Materials and Methods

### 2.1. Mouse Models of Viral Myocarditis and Dilated Cardiomyopathy

**Figure 1:**
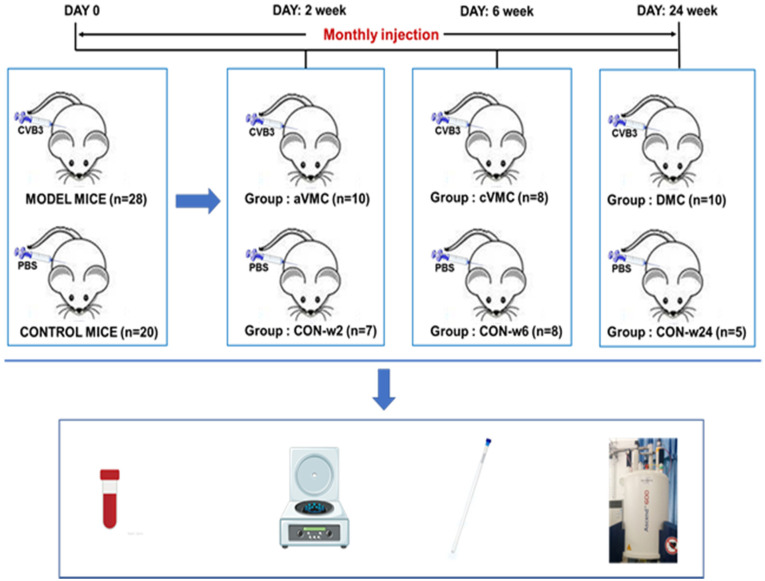
Schematic diagram of the experimental design.

Inbred male BALB/c mice (4–5 weeks of age) were supplied by Hunan Laboratory Animal Centre, Chinese Academy of Sciences (Changsha, Hunan, China). This study was performed in accordance with protocols approved by the Guangxi Medical University Animal Ethics Committee, China. All animals were maintained in a specific pathogen-free facility in the Experimental Animal Center (Guangxi Medical University, Nanning, Guangxi, China), under controlled conditions (20–24 °C and 40–70% humidity) with a 12 h light cycle, and fed standard rodent chow and water. Mice were randomly divided into MODEL mice (*n* = 28) and normal control (CON) mice (*n* = 20). The MODEL mice were divided into three groups: aVMC (*n* = 10); cVMC (*n* = 8); DCM (*n* = 10). The control mice were divided into three group: CON-w2 (*n* = 7); CON-w6 (*n* = 8); CON-w24 (*n* = 5), corresponding to the three pathological stages of aVMC, cVMC and DCM, respectively. The first day when mice were injected intraperitoneally (i.p.) was defined as day 0. Thereafter, the MODEL mice were injected monthly i.p. with 100 L of CVB3 (median tissue culture infective dose TCID50 = 10^−8^, Nancy strain) diluted in phosphate buffered saline (PBS), to establish the mouse models of aVMC, cVMC and DCM. At the same time, PBS was given monthly for the CON mice. The three groups of MODEL mice were separately sacrificed at three time points: week 2 for aVMC; week 6 for cVMC; week 24 for DCM. Correspondingly, the three groups of CON mice were also separately sacrificed at the three time points: week 2 for CON-w2; week 6 for CON-w6; week 24 for CON-w24. Both sera and hearts of the mice were removed aseptically as fresh specimens for the following experiments (Figure 1).

### 2.2. Histology

Hearts were fixed in 10% buffered formalin and stained with hematoxylin and eosin (HE) to assess inflammatory cells or Masson’s trichrome for detecting collagen deposition. Myocarditis was evaluated as the percentage of the size of the heart section (i.e., ventricles) with hematoxylin staining, necrosis, and/or fibrosis to that of the overall heart section at low power (×25 magnification) using a microscope eyepiece grid, as previously described [17]. Additionally, the collagen volume fraction (CVF) was calculated as the ratio of the area of interstitial fibrosis to the total area of connective and myocardial tissue, using the Motic Med 6.0 image (Xiamen, China) analysis software to assess the severity of myocardial fibrosis [18]. Sections were scored by at least two individuals blinded to analyzed subjects [17,18].

### 2.3. Plaque-Forming Assay

Viral titers were determined by standard plaque formation assay and expressed per organ weight (in grams). After part of the heart was weighed and homogenized, three freeze-thaw cycles and centrifuging were conducted. The HeLa cell monolayers were incubated with the supernatant for 1 h at 37 °C, 5% CO_2_, washed in PBS, and covered with 2 mL of 0.4% agar, DMEM, and 5% FCS. After 72 h of cultivation, the monolayers were fixed in para formaldehyde and stained in crystal violet, and the numbers of plaques were counted.

### 2.4. RNA Extraction and Real Time-PCR

The total RNA of homogenized heart tissues was extracted with TRIZOL Reagent (Invitrogen, Carlsbad, USA), and then reversely transcribed into cDNA with a Reverse Transcription Kit (catalog RR047A, Takara, Tokyo) according to the manufacturer’s instructions. Primers were designed by Primer Premier 5.0. The following specific primers were used: CVB3: Sense: 5′-CGGTACCTTTGTGCGCCTG T-3′; Anti-sense: 5′-CAGGCCGCCAACGCAGCC-3′. The housekeeping gene β-actin: Sense: 5′-AATTCCATCATGAAGTGTGA-3′; Anti-sense: 5′-ACTCCTGCTTGCTGATCCAC-3′. RT-PCR was performed with an initial denaturation step for 3 min at 94 °C, a three-step cycling procedure (denaturation at 94 °C for 30 s, annealing at 60 °C for 30 s, and extension at 72 °C for 60 s) for 35 cycles. The gene expressions were normalized to the level of β-actin transcripts and quantified by the CT method using the 7500 System Sequence Detection software (Applied Biosystems, Waltham, MA, USA). All reactions were performed in duplicate for each sample.

### 2.5. Sample Preparation and ^1^H-NMR Spectroscopic Analysis

Serum samples were thawed on the ice prior to NMR experiments. Then, 250 µL of the sera was mixed with 250 µL of 50 mM phosphate buffer containing 20% D_2_O (pH 7.4) in Eppendorf tubes. These tubes were centrifuged at 12,000 g for 10 min at 4 °C. Thereafter, 500 µL of the sample was transferred into 5-mm NMR tube. All ^1^H-NMR experiments were conducted on a Bruker AVANCE III HD 600 MHz spectrometer at 298 K. 1D ^1^H-NMR spectra of serum samples were acquired using the Carr-Purcell-Meiboom-Gill (CPMG) pulse sequence [RD-90°-(τ-180°-τ)*n*-ACQ] with water suppression. A fixed total spin—spin relaxation delay of 80 ms were used to attenuate broad NMR signals of slowly tumbling macromolecules with short T2 relaxation times and to retain signals of metabolites with low molecular weights. Experimental parameters were shown as follows: spectral width = 12 KHz; number of time domain data points (TD) = 64 K; relaxation delay (RD) = 4 s; acquisition time (ACQ) = 2.73 s; number of scans (NS) = 256. These NMR spectra were multiplied by an exponential function with a line-broadening factor of 0.3 Hz prior to Fourier transformation, manually phased and corrected for baseline distortion carefully. The NMR spectra of the serum samples were referenced to the methyl group of lactate (1.33). Resonances of aqueous metabolites derived from mouse sera were assigned by a combination of Chenomx NMR Suite (Version 8.3, Chenomx Inc., Edmonton, AB, Canada), Human Metabolome Data Base (HMDB, http://www.hmdb.ca/ accessed on 6 January 2022) and relevant literatures [19]. The resonance assignments were confirmed by using 2D NMR spectra, including ^1^H-^1^H TOCSY and ^1^H-^13^C HSQC spectra.

### 2.6. Multivariate Statistical Analysis

The NMR spectral data were preprocessed prior to the multivariate statistical analysis. Each NMR spectrum was segmented to regions with a width of 0.002 ppm (bin) and integrated using the MestRova software (Version 9.0, Mestrelab Research S.L., La Coruña, Spain). The spectral region was 9.00–0.20, while the region of 5.7–4.6 was excluded to eliminate distorted baseline from imperfect water saturation. The remaining integrals in each NMR spectrum were probabilistic quotient normalized [20,21]. Hierarchical clustering analysis (HCA) was conducted on the binned spectral data, which is one of multivariate statistical analyses for sample classification without training sample set. In HCA, each sample act as a separate cluster initially and the algorithm proceeds to combine them until all samples belong to one cluster. The unsupervised principal component analysis (PCA) was performed to reveal trends, highlight outliers and show clusters among the observations with the SIMCA-P+ 14.0 software (Umetrics AB, Umea, Sweden). Then, both partial least squares discriminant analysis (PLS-DA) and orthogonal signal correction partial least-squares discriminant analysis (OPLS-DA) were used to classify the samples and extract the correlated variables related to sample belongings. PLS-DA is a PLS regression where *y* is a set of binary variables describing the class belonging [22]. OPLS-DA is a derivative PLS-DA which removes the uncorrelated variables in the within-class using the orthogonal signal correction (OSC) filter [23]. Most of the variables related to the class belonging are described on the first principal component in the OPLS-DA model. Both PLS-DA and OPLS-DA were also executed with SIMCA-P+ 14.0. Thereafter, the linear classifiers were created on the basis of PLS-DA and OPLS-DA models in MATLAB (Version MATLAB2011b, MathWorks, Natick, MA, USA) (www.mathworks.com/help/stats/discriminant-analysis.html, accessed on 6 January 2022). The linear classifiers were used to verify the accuracy of classification [24].

### 2.7. Identification of Significant Metabolites and Quantitative Comparison of Metabolite Levels

We validated the robustness of the PLS-DA model by the response permutation tests (RPTs), and then identified significant metabolites with two criteria based on the validated PLS-DA models. One criterion is the variable importance in the projection (VIP) [22], and another criterion is the correlation coefficients (r) of the variables relative to the first predictive component (tp1) in the OPLS-DA model [25]. The critical values were referred to the table of critical values of correlation coefficients according to the degrees of freedom (df) which were determined as n1 + n2 − 2 with n1 and n2 as the respective numbers of samples of the two groups in the OPLS-DA model. The loading plot of the OPLS-DA model with the two criteria was reconstituted in MATLAB. Relative integrals of metabolites were used to represent relative levels of metabolites. Means and standard errors of the metabolites were calculated for each group. We conducted *t*-test to quantitatively compare metabolite levels of these three pathologic groups compared with three control groups based on the relative integrals of serum metabolites (Table 1). Quantitative comparisons of the relative levels of metabolites among these three pathological groups compared with their control groups were performed by using One-way ANOVA followed by Tukey’s multiple comparisons tests (Table S2). The univariate analysis was conducted with MATLAB Statistics Toolbox. Metabolites with the *p* value < 0.05 were identified to be differential metabolites. Metabolites with VIP > 1 from the PLS-DA model and *p* < 0.05 from the univariate analysis were identified to be characteristic metabolites.

### 2.8. Multivariate Receiver Operating Characteristic (ROC) Curve Analysis

We performed the multivariate ROC analysis to explore potential biomarkers based on the significant metabolites identified from the OPLS-DA models. We randomly selected 66.7% serum samples to perform multivariate receiver operating characteristic (ROC) curve analysis for screening of potential biomarkers in aVMC, cVMC and DCM mice relative their normal counterparts. The module of Biomarker Analysis provided by MetaboAnalyst 5.0 (https://www.metaboanalyst.ca, accessed on 6 January 2022) [26] was used to build multivariate ROC curves (Figure S4A). We selected the remaining 33.3% serum samples for multivariate ROC analysis to confirm the effectiveness of the potential biomarkers (Figure S4B). Potential biomarkers were identified by the feature ranking method with the Random Forests algorithm [27] (Figure S4C). Multivariate ROC curve analysis was performed using logistic regression algorithm for classification. The area under the ROC curve (AUC) value was used to evaluate the predictive performance of a biomarker model. The significant metabolites with AUC 0.7 and selected frequency > 0.4% were identified to be potential biomarkers for diagnosing a given pathological state. Concrete details were described in our previous work [28].

### 2.9. Metabolic Pathway Analysis

We performed the metabolic pathway analysis to identify significantly disturbed metabolic pathways (significant pathways) associated with the progression from acute VMC to DCM compared with normal controls. The metabolic pathway analysis was conducted based on relative levels of the metabolites using the module of Pathway Analysis in MetaboAnalyst 5.0 [26]. The metabolic pathway analysis well integrates the metabolite set enrichment analysis (MESA) and pathway topology analysis. As a metabolomic version of the popular gene set enrichment analysis [29], MSEA has its own collection of metabolite set libraries with user-friendly web-interfaces [26]. As a novel way to identify biologically meaningful metabolic patterns closely associated with metabolite levels, MESA assesses whether a group of functionally related metabolites are significantly enriched by calculating statistical *p* values, which has the potential to identify “subtle but consistent” changes among a group of related metabolites. On the other hand, metabolic alterations occurring in important nodes of the metabolic network would potentially trigger significant impacts on the metabolic pathway than those occurring in marginal or relatively isolated nodes. We performed the pathway topology analysis through computing pathway impact values (PIV) with relative-betweenness centrality arithmetic. Significantly disturbed metabolic pathways were identified with pathway impact values > 0.2 and *p* < 0.05, using the Pathway Analysis module provided by MetaboAnalyst 5.0.

## 3. Results

### 3.1. Viral Myocarditis and Dilated Cardiomyopathy Induced by CVB3 in Mice

**Figure 2:**
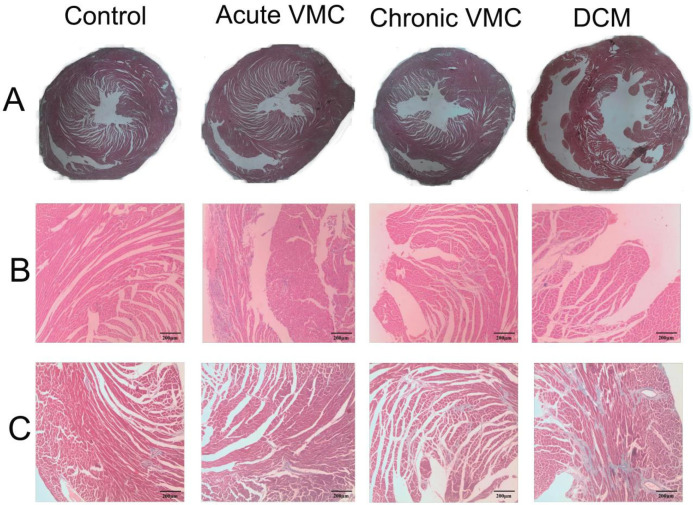
Representative histopathological images of myocardial tissues derived from the established models of aVMC, cVMC and DCM mice and the normal control mice (CON). (**A**) Histopathological images of the myocardial tissues; (**B**) Tissue sections stained with hematoxylin-eosin (×400); (**C**) Tissue sections stained with Masson (×400).

Hearts were cut longitudinally and assessed histologically for dilation at low power. In the control groups (CON-w2, CON-w6 and CON-w24), HE staining of heart tissues did not show profound cardiac necrosis and inflammatory infiltration (Figure 2A,B). Moreover, interstitial fibrosis did not display significant differences between the three control groups, as evaluated by the calculated CVF (%) values (Figure 2C). Furthermore, neither cavity dilatation nor decreased wall thickness in ventricles was observed among the three control groups. In the three CVB3-infected groups (aVMC, cVMC and DCM), the heart tissues showed significant degeneration and necrosis of cardiomyocytes, inflammatory infiltration, collapse of cardiac muscle fibers, and little fibrosis around the necrosis (Figure 2A,B). During the pathological progression, the aVMC group exhibited the most significant cardiac inflammatory infiltration, which was thereafter gradually declined. Fibrosis was gradually increased over the course of experimental time in the CVB3-infected groups (Figure 2C). No inflammatory infiltration but massively diffused fibrosis was observed in the DCM group. Moreover, obviously cavity dilation and decreased wall thickness of ventricles were observed in the DCM group, but not in aVMC and cVMC groups. These data indicated that the mouse models of aVMC (week 2), cVMC (week 6) and DCM (week 24) were successfully established by inducing with CVB3 infection. To evaluate the effect of monthly CVB3 injection on metabolic profiles of the mice, we measured cardiac CVB3 mRNA levels and viral titers by using RT-PCR and standard plaque formation, respectively. The cardiac CVB3 mRNA was highly expressed on week 2 in the CVB3-infected group, then gradually decreased (aVMC: 4.3 ± 2.2, cVMC: 2.1 ± 0.5, DCM: 2.5 ± 0.7). Moreover, the viral titers in the three pathological groups showed the similar changing tendency along the course of the experimental time: aVMC, (1.8 ± 0.5) × 10^6^; cVMC, (1.2 ± 0.3) × 10^2^; DCM, (1.1 ± 0.4) × 10^2^. Given that the cardiac viral was not gradually increased by monthly CVB3 injection, metabolic disorders associated with the chronic VMC and DCM states mostly resulted from the pathological progression rather than the monthly CVB3 injection.

### 3.2. Metabolic Alterations in CVB3-Infected Groups Compared with Controls

**Figure 3:**
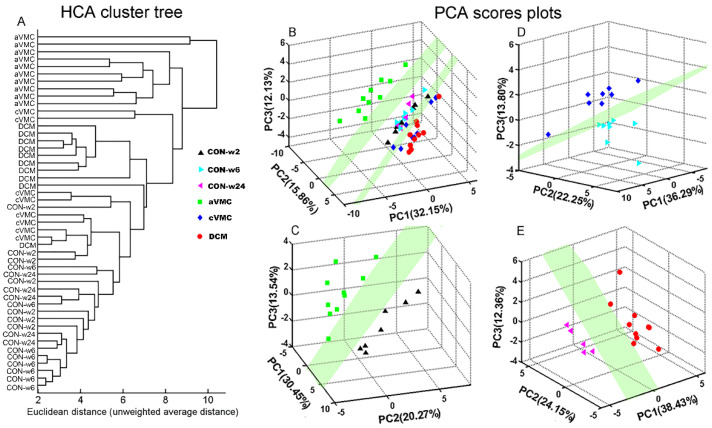
Multivariate analyses for 1D ^1^H-NMR spectra recorded on sera derived from six groups of mice (CON-w2, CON-w6, CON-w24, aVMC, cVMC, DCM). (**A**) HCA clustering tree for the six groups; (**B**) PCA scores plot for the six groups; (**C**–**E**) PCA scores plots for aVMC and CON-w2 (**C**); for cVMC and CON-w6 (**D**); for DCM and CON-w24 (**E**). Each point represents a serum sample derived from an individual mouse.

**Figure 4:**
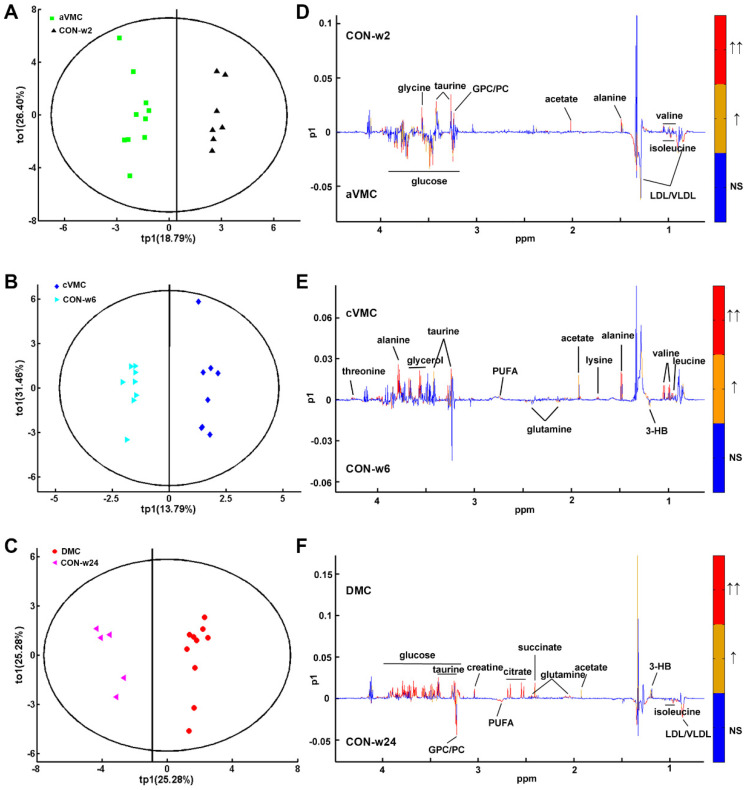
OPLS-DA analyses of the mouse sera to identify significant metabolites significantly responsible for distinguishing metabolic profiles between three pathologic groups (aVMC, cVMC, DCM)) and control groups (CON-w2, CON-w6, CON-w24). (**A**–**C**) OPLS-DA scores plots; (**D**–**F**) the corresponding OPLS-DA loading plots. The red color indicates that the variables are very significant (|r| > 0.482 in (**D**), |r| > 0.497 in (**E**), |r| > 0.514 in (**F**); VIP > 1); orange indicates that the variables are significant (0.349 <|r| < 0.482 in (**D**), 0.355 <|r| < 0.492 in (**E**), 0.361 <|r| < 0.514 in (**F**); VIP > 1); blue indicates that the variables are insignificant (NS).

Figure S1 illustrates typical 1D 1H-NMR spectrum recorded on the serum derived from an aVMC mouse. Totally, 28 metabolites were identified in the NMR spectrum (Table S1). To acquire overall metabolic information and examine metabolic profiles of the six groups of sera, we performed unsupervised HCA and PCA analyses on the NMR data sets of three CVB3-infected groups (aVMC, cVMC and DCM) and their control counterparts (CON-w2, CON-w6 and CON-w24). The three control groups of sera displayed indistinguishable metabolic profiles (Figure 3A,B). However, the three infected groups exhibited distinctly different metabolic profiles from the corresponding CON groups, i.e., aVMC vs. CON-w2, cVMC vs. CON-w6, DCM vs. CON-w24 (Figure 3C–E). To maximize metabolic distinctions between the infected groups and the normal control groups, we conducted the PLS-DA analyses on the NMR data sets. Figure S2 exhibits the scores plots of the PLS-DA models built with the first two predictive principal components (tp1 and tp2). The linear classifier boundaries in these plots illustrate that the CBV3-infected mice were metabolically distinguished clearly from their normal counterparts. Furthermore, we performed response permutation tests (RPTs) with 200 cycles to validate the robustness of the PLS-DA models, which showed that these three models were not overfitting (Figure S3). Furthermore, we constructed six pairwise OPLS-DA models with tp1 based on the NMR data sets of the sera (Figure 4). The OPLS-DA scores plots show distinct metabolic separations between the CVB3-infected groups and their normal counterparts (Figure 4A–C). Totally, 9, 11 and 12 significant metabolites were identified for aVMC vs. CON-w2, cVMC vs. CON-w6, DCM vs. CON-w24 from the OPLS-DA loading plots, respectively (Figure 4D–F). In addition, we conducted *t*-test to quantitatively compare metabolite levels of the three pathologic groups compared with the three control groups based on the relative integrals of serum metabolites (Table 1). Totally, 7, 10 and 15 differential metabolites were identified for pair-wise comparisons of aVMC vs. CON-w2, cVMC vs. CON-w6, DCM vs. CON-w24, respectively. Finally, we identified characteristic metabolites with VIP > 1 and *p* < 0.05 (Table S3). Totally, 7, 8 and 11 characteristic metabolites were identified for pair-wise comparisons of aVMC vs. CON-w2, cVMC vs. CON-w6, DCM vs. CON-w24, respectively.

### 3.3. Levels of the Metabolites Were Changed in the Three Pathological States

#### 3.3.1. Amino Acid Metabolism

Overall, the pathological mice showed significantly changed levels of three branch chain amino acids (BCAAs) compared with CON mice, including isoleucine, leucine and valine (Table 1). aVMC mice displayed distinctly decreased valine, and almost unchanged isoleucine and leucine. cVMC mice showed up-regulated levels of the three BCAAs, and DCM mice exhibited remarkably decreased leucine. Moreover, the three pathologic groups showed relative stable levels of threonine compared with their counterparts. Furthermore, glycine was markedly decreased in aVMC mice but remarkably increased in DCM mice without observable change in cVMC mice. In addition, lysine was profoundly increased in cVMC mice but not significantly altered in aVMC and DCM mice. Alanine was not significantly changed in the three pathologic groups. Taurine was markedly decreased in aVMC mice and significantly increased in cVMC and DCM mice. Furthermore, glutamine was decreased in cVMC mice and obviously increased in DCM mice without observable change in aVMC mice.

#### 3.3.2. Carbohydrate Metabolism

Compared with CON mice, aVMC and cVMC mice exhibited reduced levels of glucose, but DCM mice show a significantly enhanced level of glucose. Moreover, two TCA-related metabolites (succinate and citrate) were significantly increased in DCM mice without observable changes in aVMC and cVMC mice. Acting as one of the terminal metabolites of glycolysis, lactate was slightly increased in DCM mice, but kept unchanged in aVMC and cVMC mice. Furthermore, acetate was markedly decreased in aVMC mice but slightly increased in cVMC and DCM mice. In addition, creatine was somewhat increased in DCM mice without observable changes in aVMC and cVMC mice.

#### 3.3.3. Lipid Metabolism

Relative to CON mice, serum levels of LDL and VLDL were remarkably increased in aVMC mice and distinctly decreased in DCM mice, without detectable change in cVMC mice. Moreover, PUFA was slightly increased in cVMC mice and profoundly decreased in DCM mice, but not significantly changed in aVMC mice. Furthermore, 3-hydroxybutyrate was slightly increased in DCM mice, but remained virtually unchanged in aVMC and cVMC mice. Additionally, cVMC and DCM mice displayed significantly up-regulated glycerol levels, while aVMC mice showed an unchanged glycerol level. 

#### 3.3.4. Choline phosphorylation metabolism

After the CVB3-infection, the choline phosphorylation metabolism of mice became disorder in sera. GPC was slightly decreased in aVMC and DCM mice but not observably changed in cVMC mice.

### 3.4. Potential Biomarkers in the Progression from Acute VMC to DCM

**Figure 5:**
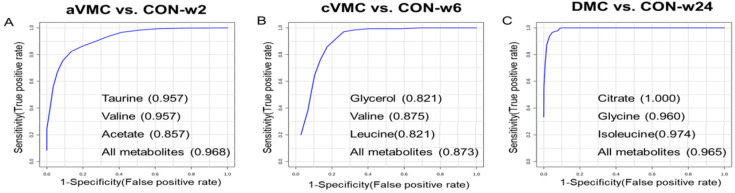
Multivariate ROC analysis of mouse sera for identifying potential biomarkers. (**A**) Taurine, valine and acetate identified from the ROC analysis of aVMC vs. CON-w2. (**B**) Glycerol, valine and leucine identified from cVMC vs. CON-w6. (**C**) Citrate, glycine and isoleucine identified from DMC vs. CON-w24.

We conducted multivariate ROC analysis to decide potential biomarkers based on the identified significant metabolites. The screened significant metabolites were ranked by frequencies of being selected during Monte-Carlo cross validation performed with the Random Forests algorithm (Figure S4). The top three significant metabolites were identified to be potential biomarkers with AUC 0.7 and selected frequency 0.4 (Figure 5). The aVMC stage showed large AUCs of the ROC curves built by using either only one of the following metabolites or their combination: 0.957 for taurine; 0.957 for valine; 0.857 for acetate; 0.968 for these three metabolites. The cVMC stage also displayed large AUCs by using the following metabolites: 0.821 for glycerol; 0.875 for valine; 0.821 for leucine; 0.873 for these three metabolites. Finally, the DCM stage exhibited large AUCs too by using the following three metabolites: 1.000 for citrate; 0.960 for glycine; 0.974 for isoleucine; 0.965 for these three metabolites.

### 3.5. Significantly Disturbed Metabolic Pathways in the Three Pathological Stages

**Figure 6:**
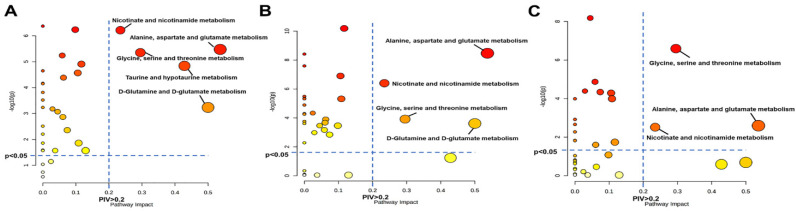
Significantly disturbed metabolic pathways identified from pathway analyses based on serum levels of metabolites. (**A**) aVMC vs. CON-w2; (**B**) cVMC vs. CON-w6; (**C**) DCM vs. CON-w24. Pathway impact values (PIVs) were calculated from pathway topology analysis, and *p* values were computed from metabolite set enrichment analysis. Significantly disturbed metabolic pathways were identified with pathway impact values > 0.2 and *p* values < 0.05, using the Pathway Analysis module provided by MetaboAnalyst 5.0 webserver.

We performed metabolic pathway analysis to select significantly disturbed metabolic pathways (significant pathways) in the progression of VMC into DCM based on metabolite levels in aVMC, cVMC and DCM groups relative to their normal counterparts. Two criteria of PIV > 0.2 and *p* < 0.05 were used to identify significant pathways (Figure 6). The aVMC stage showed three significant pathways: glycine, serine and threonine metabolism; pyruvate metabolism; taurine and hypotaurine metabolism (Figure 6A). The cVMC stage displayed only one significant pathway: taurine and hypotaurine metabolism (Figure 6B). The DCM stage exhibited four significant pathways: glycine, serine and threonine metabolism; pyruvate metabolism; taurine and hypotaurine metabolism; glycerolipid metabolism (Figure 6C). The three pathological stages shared a significant pathway (taurine and hypotaurine metabolism) with the highest PIV values.

## 4. Discussion

The progression from acute VMC to DCM is related to several pathological stages. Until now, few metabolomic analyses have been conducted to explore metabolic profiles during this progression. In the present study, we established three mouse models of VMC progressing into DCM by infecting the mice with CVB3. Cardiac pathological examination showed that these three models reflected three pathological stages: acute VMC (aVMC), chronic VMC (cVMC) and DCM, similar to those of VMC progressing into DCM in human. We performed NMR-based metabolomic analyses of these three pathological stages, which showed distinctly altered metabolic profiles, dramatically changed metabolite levels, and significantly disturbed metabolic pathways compared with their normal counterparts.

As the most metabolically demanding organ in the body, the heart relies preferentially on fatty acid metabolism to sustain sufficient ATP supply. Furthermore, the heart possesses a unique capability to metabolize a variety of substrates besides fatty acids, such as carbohydrates (glucose and lactate), ketone bodies and amino acids. This capability allows the heart to sustain constant contractile function [30]. Our work demonstrates that several energy-related metabolic pathways are significantly disturbed in the progression from acute VMC to DCM, including glycine, lysine, alanine and threonine metabolism and Krebs cycle. Obviously, glycine was decreased in aVMC but increased in DCM, which was identified to be a potential biomarker in the DCM stage. Furthermore, we revealed that glycine, serine and threonine metabolism is the significantly disturbed metabolic pathways in the DCM stage. Previously, Maneikyte et al. showed that glycine can protect the heart against chemotherapy- induced injury during the treatment of colorectal liver metastasis, by preserving the left ventricle ejection fraction (LVEF) and reducing the levels of fibrosis and apoptosis [31]. Thus, it seems that glycine might contribute to protecting DCM from heart remodeling.

In the DCM stage, the decreased levels of PUFA and LDL/VLDL indicated down-regulated mitochondrial oxidation of fatty acids, the most important pathway for cardiac energy supply. In contrast, 3-HB, the most important ketone, was increased dramatically in the DCM stage. It was previously reported that cardiac mitochondrial oxidative metabolism and glucose oxidation are suppressed in an energy starved heart in cardiomyopathy and heart failure [32]. Thus, the up-regulated level of 3-HB might be an adaptive response to lessen the severity of heart failure and increase energy supply in cardiomyopathy [32,33].

Glycerolipid metabolism was identified to a significantly disturbed metabolic pathway in the DMC stage. Up-regulated levels of glycerol were observed in the cVMC and DCM stages. Previous study suggested that glycerol release acts as an indicator of arrhythmias in ischemic myocardium [34]. Further study should be performed to exam whether the up-regulated glycerol levels in the cVMC and DCM stages are associated with arrhythmias.

Notably, carbohydrate metabolism was promoted dramatically in the DCM stage, with enhanced levels of creatine, acetate, glucose, lactate, succinate and citrate. This suggests that the impaired carbohydrate metabolism might contribute to the promoted supply of energy in the DCM stage. Furthermore, our work identified acetate to be a potential biomarker in the aVMC stage, and citrate in the DCM stage. Magnusson et al. have applied isotopic tracers including ^11^C-acetate, ^15^O-water and ^11^C-HED as risk markers of positron emission tomography, with regard to non-sustained ventricular tachycardia (NSVT) in hypertrophic cardiomyopathy [35]. NSVT provides a marker for sudden cardiac death. It remains to be elucidated whether the profoundly enhanced level of citrate in the DCM stage is associated with NSVT and sudden cardiac death.

As described above, both glucose metabolism and fatty acid metabolism play important roles in the DCM stage. Note that the levels of branched chain amino acids are also significantly altered in this stage [36]. As is known, BCAAs (valine, leucine and isoleucine) also play crucial roles in many metabolic pathways such as protein synthesis. Our study showed that valine was significantly decreased in the aVMC stage, while leucine was markedly decreased in the DCM stage potentially due to anorexia-induced decrease in food intake. However, all of BCAAs were increased in the cVMC stage. Kimura et al. found that in patients with nonischemic dilated cardiomyopathy (NIDCM), the ratio of BCAAs number to total amino acid residues number (termed BCAAs/total AAs) is positively correlated with LVEF and negatively correlated with brain natriuretic peptide (BNP) [37]. The group of NIDCM patients with the low ratio of BCAAs/total AAs has a lower cardiac event-free rate. It seems that this ratio could serve as a useful predictor for future cardiac events in NIDCM patients. Notably, a previous study performed in ischemia/reperfusion (I/R) mouse has demonstrated that BCAAs exacerbate myocardial I/R vulnerability through fatty acid oxidation [36]. Those results suggest that BCAAs could either be beneficial or be harmful in different cardiac disease states. Further study need to be conducted to exploit the potential roles of BCAAs in the progression of VMC in to DCM.

Furthermore, our study identified taurine to be a potential biomarker in the aVMC stage, and taurine mechanism to be a significantly disturbed metabolic pathway in these three pathological stages. Relative to the CON stages, taurine was decreased in the aVMC stage, but increased in the cVMC and DCM stages. As a nonessential amino acid, taurine shows significant beneficial effects in cardiovascular diseases [38], attributing to its modulation of Ca^2+^ homeostasis and its antioxidant properties [39]. However, short-term exposure to taurine could increase intracellular levels of Na^+^ and Ca^2+^ [39], which would promote stronger contraction of blood vessels. Contrarily, long-term exposure to taurine could decrease intracellular levels of Na^+^ and Ca^2+^ [39]. Thus, the increased taurine in the cVMC and DCM stages might contribute to decrease in the contractility of blood vessels, which needs to be confirmed by future study.

In addition, our data showed that glutamine was slightly decreased in the cVMC stage but significantly increased in the DCM stage. Glutamine metabolism is usually involved in oxidation stress. A previous study has demonstrated that glutamine can protect cardiac cells against the acute cantharidin-induced cardiotoxicity [40]. Thus, the modulation of glutamine levels in the VMC and DCM stages might be of benefit to protection of myocardial cells.

Previous studies have shown that glycerophosphocholine (GPC) can reserve mitochondrial respiration, reduce ischemia-induced oxidative stress and decrease radical production [41,42,43]. Furthermore, cytoprotective effects of short-term GPC treatment have been observed in cardiac myocytes [44], including physiological balance of ROS production and cell viability. In our study, the levels of GPC were down-regulated in the aVMC and DCM stages. It is thereby expected that GPC treatment in the aVMC and DCM stages might reduce oxidative stress and enhance cell viability. Further studies are required to confirm this expectation.

## 5. Conclusions

We have performed NMR-based metabolomic analyses to explore the progression from acute VMC to DCM on established mouse models mimicking three pathological stages (aVMC, cVMC, DCM). The progression of VMC into DCM shows dramatically cardiac metabolic remodeling. The branched chain amino acids metabolism is disordered, implying impaired protein synthesis. Furthermore, several metabolic pathways are significantly disturbed, including taurine and hypotaurine metabolism, glycerolipid metabolism, glycine, serine and threonine metabolism, indicating impaired antioxidation and antiapoptotic properties as well as disordered energy metabolism. In this study, we have not measured expressions and activities of regulatory enzymes involved in the identified significant pathways. Such work should be carried out in the future to confirm that these pathways are associated with the progression of VMC into DCM.

Furthermore, compared with the CON stages, we identified several potential biomarkers for metabolically discriminating the progression from acute VMC to DCM: taurine, valine and acetate for the aVMC stage; glycerol, valine and leucine for the cVMC stage; citrate, glycine and isoleucine for the DCM stage. Further works are required to comprehensively evaluate potencies of these potential biomarkers for clinical diagnoses based on large-scale samples. Our results provide new insights into the metabolic mechanisms underlying these three pathological stages, and may be beneficial to exploitation of potential biomarkers for clinically diagnosing and monitoring the progression of VMC into DCM.

## Figures and Tables

**Table 1:** Relative levels of metabolites calculated from 1D ^1^H-NMR spectra of sera derived from the six groups of mice.

	CON-w2	CON-w6	CON-w24	aVMC	cVMC	DCM
**Amino acid metabolism**						
leucine	0.814 ± 0.114	0.834 ± 0.060	0.866 ± 0.102	0.866 ± 0.123	0.917 ± 0.063↑	0.677 ± 0.090↓↓
isoleucine	0.148 ± 0.024	0.144 ± 0.015	0.153 ± 0.022	0.125 ± 0.050	0.173 ± 0.028↑	0.124 ± 0.031
valine	0.397 ± 0.035	0.396 ± 0.034	0.420 ± 0.083	0.267 ± 0.078↓↓↓	0.514 ± 0.085↑↑	0.363 ± 0.088
threonine	0.256 ± 0.077	0.227 ± 0.061	0.213 ± 0.056	0.261 ± 0.094	0.248 ± 0.066	0.220 ± 0.052
glycine	0.333 ± 0.020	0.339 ± 0.067	0.327 ± 0.013	0.284 ± 0.037↓↓	0.346 ± 0.041	0.448 ± 0.087↑↑
lysine	0.508 ± 0.071	0.499 ± 0.057	0.505 ± 0.051	0.465 ± 0.138	0.589 ± 0.070↑	0.520 ± 0.083
alanine	0.314 ± 0.020	0.351 ± 0.044	0.348 ± 0.034	0.285 ± 0.055	0.372 ± 0.046	0.401 ± 0.061
taurine	1.622 ± 0.192	1.511 ± 0.103	1.537 ± 0.104	1.246 ± 0.148↓↓↓	1.641 ± 0.096↑	1.926 ± 0.143↑↑↑
glutamine	0.767 ± 0.142	0.723 ± 0.055	0.761 ± 0.091	0.906 ± 0.245	0.647 ± 0.072↓	1.007 ± 0.168↑↑
**Carbohydrate metabolism**						
creatine	0.384 ± 0.070	0.364 ± 0.039	0.382 ± 0.074	0.320 ± 0.074	0.326 ± 0.048	0.498 ± 0.092↑
acetate	0.231 ± 0.057	0.223 ± 0.072	0.189 ± 0.037	0.154 ± 0.025↓↓	0.311 ± 0.084↑	0.282 ± 0.073↑
glucose	3.421 ± 0.425	3.066 ± 0.353	3.342 ± 0.113	2.971 ± 0.399↓	2.596 ± 0.285↓	3.855 ± 0.324↑↑↑
lactate	4.266 ± 0.772	4.529 ± 0.389	4.632 ± 0.767	4.356 ± 0.475	4.215 ± 0.511	5.468 ± 0.513↑
succinate	0.195 ± 0.108	0.196 ± 0.075	0.160 ± 0.062	0.221 ± 0.076	0.166 ± 0.025	0.328 ± 0.128↑
citrate	0.224 ± 0.060	0.253 ± 0.042	0.243 ± 0.033	0.242 ± 0.078	0.241 ± 0.029	0.586 ± 0.158↑↑↑
**Lipid metabolism**						
LDL/VLDL	7.579 ± 0.462	7.941 ± 0.724	7.240 ± 0.601	8.955 ± 0.801↑↑↑	8.696 ± 1.911	5.607 ± 0.546↓↓↓
PUFA	2.254 ± 0.382	2.109 ± 0.321	2.086 ± 0.204	2.216 ± 0.471	2.495 ± 0.355↑	1.526 ± 0.284↓↓
3-HB	0.440 ± 0.109	0.460 ± 0.057	0.482 ± 0.161	0.462 ± 0.100	0.405 ± 0.161	0.760 ± 0.247↑
glycerol	1.430 ± 0.133	1.478 ± 0.239	1.449 ± 0.050	1.399 ± 0.378	1.700 ± 0.113↑	2.098 ± 0.320↑↑↑
**Choline phosphorylation metabolism**						
GPC	4.104 ± 0.377	4.459 ± 0.700	4.269 ± 0.351	3.470 ± 0.538↓	4.544 ± 0.440	3.712 ± 0.363↓

**Note:** ↑↑↑/↓↓↓, ↑↑/↓↓, ↑/↓ mean that the changes of relative metabolite levels in the mouse models are highly significant (*p* < 0.001), very significant (*p* < 0.01), significant (*p* < 0.05) compared with those in the corresponding normal mice (aVMC vs. CON-w2, cVMC vs. CON-w6, DCM vs. CON-w24). ↑ and↓ denote significant increase and significant decrease, respectively.

## Data Availability

Data is contained within the article or supplementary material.

## Supplementary Materials

The following supporting information can be downloaded at: https://www.mdpi.com/article/10.3390/biom12010112/s1, Figure S1: A typical 1D 1H-CPMG NMR spectrum of the serum derived from the aVMC mouse (4.7–0.6 ppm, 9.0–6.0 ppm); Figure S2: PLS-DA scores plots of the sera derived from the mouse models; Figure S3: Validation plots of the PLS-DA models for the sera generated from the permutation tests that were randomly permuted 200 times with the first two components; Figure S4: Multivariate ROC analysis of mouse sera for identifying important metabolites; Table S2: Quantitatively comparisons of metabolite levels during the process from acute VMC to DMC based on relative integrals of metabolites calculated from 1D 1H-NMR spectra of sera; Table S3: Characteristic metabolites were determined by a combination of significant metabolites identified from the OPLS-Da (VIP > 1) and differential metabolites identified from the univariate analyses (*p* < 0.05).

## Sample Availability

Samples of the NMR experiments are available from the authors.

## Abbreviations

The following abbreviations are used in this manuscript:

VMC	Viral myocarditis
DCM	Dilated cardiomyopathy
CVB3	Coxsackievirus B3
aVMC	Acute viral myocarditis
cVMC	Chronic viral myocarditis
CON	Normal controls
HF	Heart failure
PBS	Phosphate buffered saline
HCA	Hierarchical clustering analysis
PCA	Principal component analysis
RPTs	Response permutation tests
ROC	Receiver operating characteristic
PIV	Pathway impact values
LVEF	Left ventricle ejection fraction
BCAAs	Branch chain amino acids
PLS-DA	Partial least squares discriminant analysis
OPLS-DA	Orthogonal signal correction partial least-squares discriminant analysis
