# Supplementary material for: NMR-Based Metabolomic Analysis of Sera in Mouse Models of CVB3-Induced Viral Myocarditis and Dilated Cardiomyopathy
Source: Biomolecules. 2022 Jan 11;12(1):112. doi: 10.3390/biom12010112 (PMC8773787; doi:10.3390/biom12010112)
Supplement: Supplementary file 1 [file biomolecules-12-00112-s001.zip › biomolecules-1523347-supplementary.pdf]

NMR-based metabolomic analysis of sera in mouse models of CVB3-induced viral myocarditis and dilated cardiomyopathy

Qing Kong, Jinping Gu, Ruohan Lu, Caihua Huang, Xiaomin Hu, Yanli Liu, Xingcui Gao, Weifeng Wu, Donghai Lin

4 Supplementary Figures;

3 Supplementary Tables.

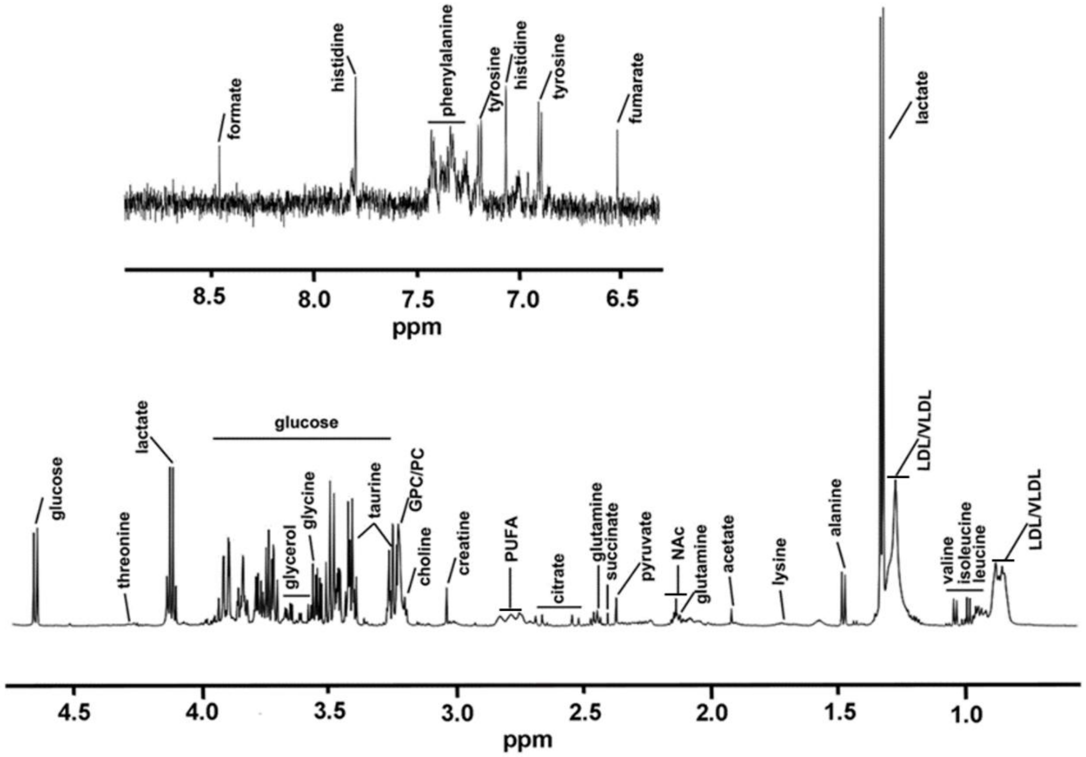

Fig. S1. A typical 1D ^{1}H-CPMG NMR spectrum of the serum derived from the aVMC mouse (4.7-0.6 ppm, 9.0-6.0 ppm).

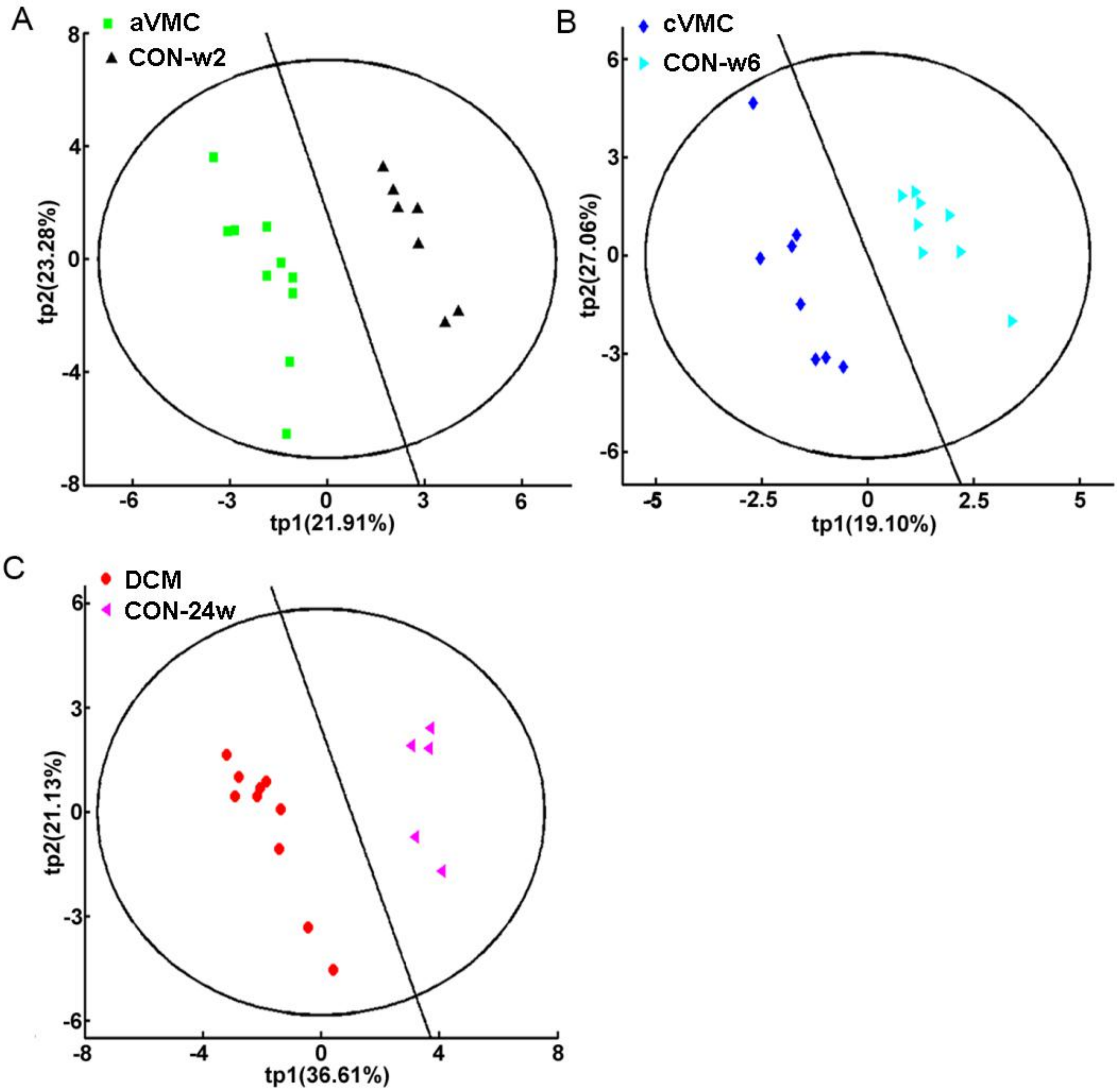

Fig. S2. PLS-DA scores plots of the sera derived from the mouse models. (A) aVMC *vs.*CON-w2; (B) cVMC *vs.*CON-w6; (C) DCM *vs.*CON-w24.

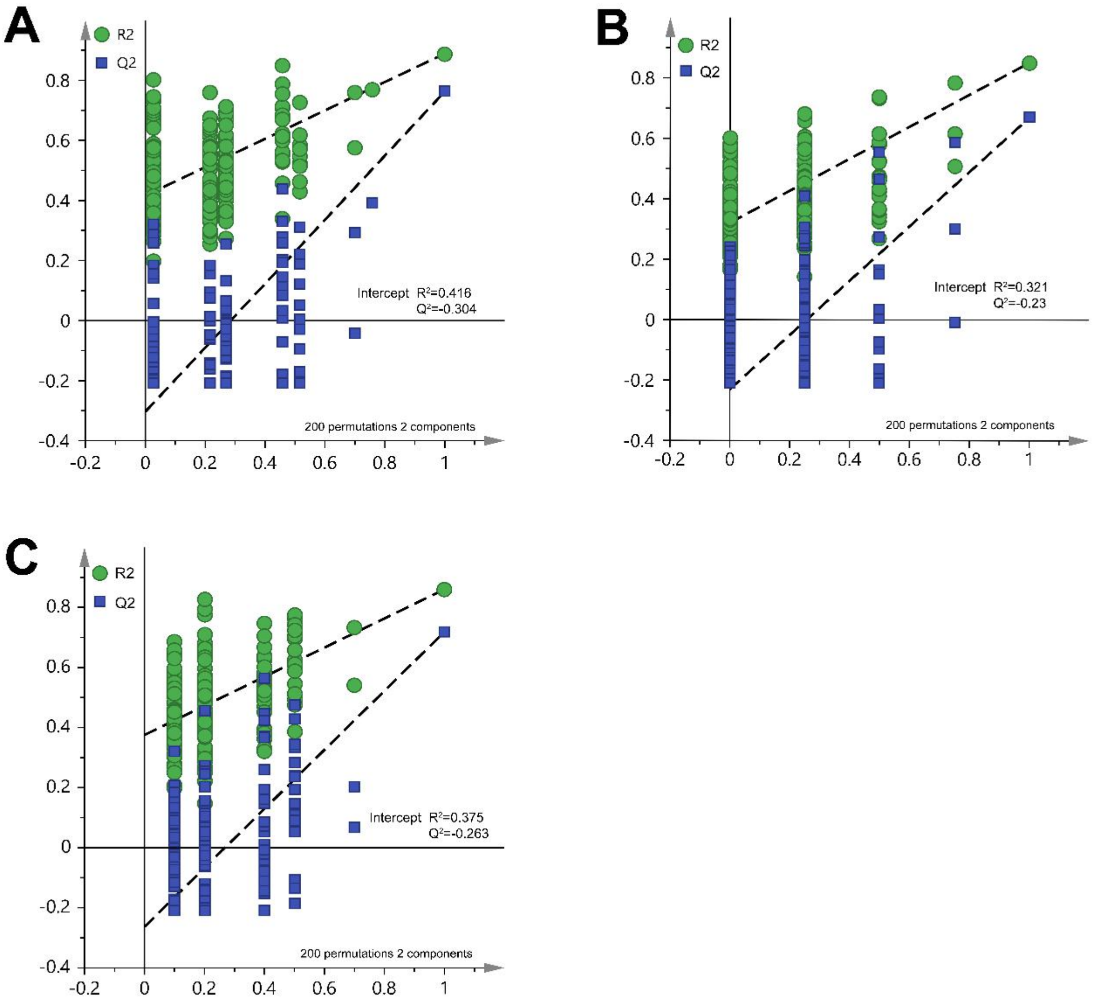

Fig. S3. Validation plots of the PLS-DA models for the sera generated from the permutation tests that were randomly permuted 200 times with the first two components. The green circle is R2 (cum), denoting the explained variance of the model. The blue square is Q2 (cum), standing for the predictive ability of the model. (A) aVMC *vs.* CON-w2; (B) cVMC *vs.* CON-w6; (C) DCM *vs.* CON-w24.

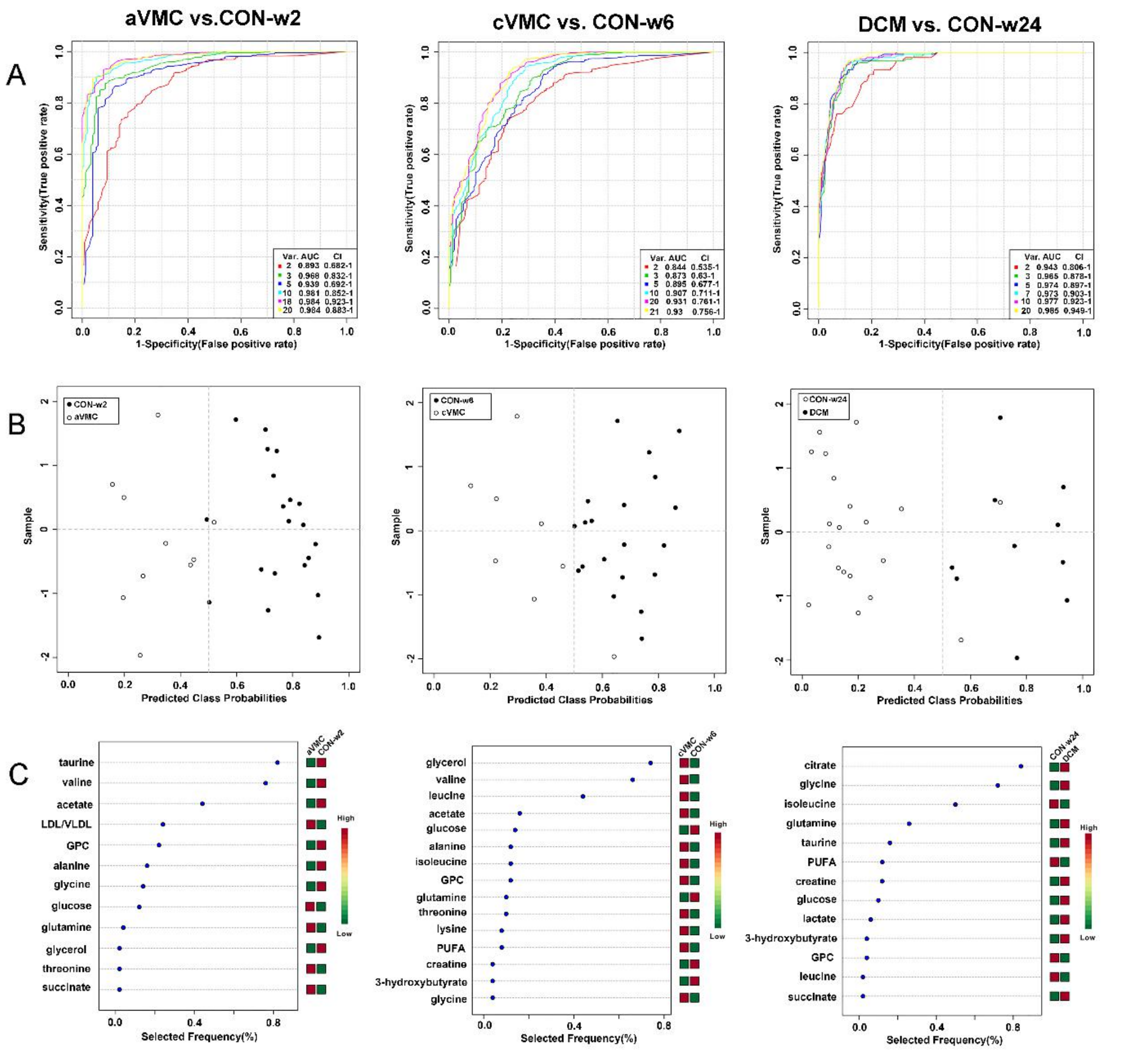

Fig. S4. Multivariate ROC analysis of mouse sera for identifying important metabolites. (A) ROC curves from different models built with different numbers of metabolites by using Monte-Carlo cross validation; (B) Predicted class probability for each sample using the base classifier (based on AUC of the ROC curve built with three metabolites). (C) Important metabolites ranked by the frequencies of being selected during cross validation.

Table S1. Resonance assignments of metabolites in 1D ^{1}H-NMR spectra of mouse sera.

Num.	Metabolite	δ ^{1}H (ppm) and multiplicity	Moieties
1	LDL/VLDL	0.89(bar),1.28(bar)	
2	leucine	0.96(d), 0.97(d), 1.69(m), 1.70(m), 1.73(m), 3.73(m)	α-CH_3, α-CH_3, γ-CH, β-CH_2, α-CH
3	isoleucine	0.94(t), 1.01(d), 1.21(m), 1.42(m), 2.00(m), 3.67(d)	δ-CH_3, γ-CH_3, half γ-CH_2, half γ-CH_2, β-CH, α-CH
4	valine	0.99(d), 1.05(d), 2.26(m), 3.60(d)	γ-CH_3, γ-CH_3, β-CH, α-CH
5	3-HB	1.197(d), 2.314(m), 2,394(m), 4.142(m)	γ-CH_3, β-CH_2, γ-CH
6	lactate	1.33(d), 4.11(q)	β-CH_3, α-CH
7	threonine	1.30(d), 3.58(d), 4.24(m)	γ-CH_2, β-CH
8	alanine	1.47(d), 3.78(q)	β-CH_3, α-CH
9	lysine	1.43(m), 1.49(m), 1.70(m), 1.91(m), 3.02(t), 3.75(t)	half γ-CH_2, half γ-CH_2, δ-CH_2, β-CH_2, ε-CH_2, α-CH
10	acetate	1.91(s)	CH_3
11	succinate	2.41(s)	CH
12	glutamine	2.13(m), 2.45(m), 3.77(t)	γ-CH_2, β-CH_2, α-CH
13	citrate	2.51(d),2.68(d)	β-CH_2
14	PUFA	5.23-5.38(bar)	
15	creatine	3.04(s), 3.93(s)	N-CH_3, α-CH_2
16	GPC/PC	3.23(s), 3.60(dd), 3.68(dd),	N-$(CH_3)_3$, half $^{1}CH_2$, $^{2}CH_2$,

		3.87(m), 3.94(m), 4.33(m)	half $^{1}CH_2$, half $^{3}CH_2$, half $^{3}CH_2$, $^{1}CH_2$
17	taurine	3.24(t), 3.41(t)	$^{1}CH_2$, $^{2}CH_2$
18	glycine	3.57(s)	α-CH_2
19	glycerol	3.56(dd), 3.64(dd), 3.77(m)	half-$^{1}CH_2$, half-$^{3}CH_2$, ^{3}CH
20	glucose	5.22(d), 4.63(d), 3.9(dd), 3.72(m), 3.48(m), 3.22(dd)	β(H_2, H_3, H_5), α (H_2, H_3, H_6)
21	N-acetyl	2.02-2.06(bar)	CH^3
22	pyruvate	2.38(s)	α-CH_3
23	choline	3.21(s), 3.51(dd), 4.04(t)	N-$(CH_3)_3$, α-CH_2, CH_2OH
24	fumarate	6.51(s)	CH
25	tyrosine	3.05(dd), 3.19(dd), 6.92(d),7.19(d)	half β-CH_2, half β-CH_2, β-CH, α-CH
26	phenylalanine	3.12(dd), 3.30(dd), 3.99(dd), 7.33(d),7.37(t), 7.43(t)	α-CH, half β-CH_2, half β-CH_2, α-CH, β-CH, γ-CH
27	histidine	7.06(s),7.85(s)	^{5}CH, ^{2}CH
28	formate	8.46（s）	CH

Note: s (singlet), d (double), t (triplet), q (quartet), m (multiple), dd (double of double).

Table S2. Quantitatively comparisons of metabolite levels during the process from acute VMC to DMC based on relative integrals of metabolites calculated from 1D ^{1}H-NMR spectra of sera.

	Tukey's multiple comparisons test			One-way ANOVA	
	aVMC vs. CON-w2	cVMC vs. CON-w6	DCM vs. CON-w24	F	p
**Amino acid metabolism**					
leucine	ns	ns	ns	6.884	9.003×10^{-5}
isoleucine	ns	ns	ns	2.824	2.752×10^{-2}
valine	**	*	ns	10.942	8.748×10^{-7}
threonine	ns	ns	ns	0.617	6.875×10^{-1}
glycine	*	ns	*	9.686	3.323×10^{-6}
lysine	ns	ns	ns	1.833	1.272×10^{-1}
alanine	ns	ns	ns	6.952	8.259×10^{-5}
taurine	*	ns	***	25.703	8.790×10^{-12}
glutamine	ns	ns	*	6.474	1.520×10^{-4}
**Carbohydrate metabolism**					
creatine	ns	ns	ns	8.228	1.742×10^{-5}
acetate	ns	ns	ns	7.392	4.772×10^{-5}
glucose	ns	ns	**	14.081	4.283×10^{-8}
lactate	ns	ns	ns	6.445	1.578×10^{-4}
succinate	ns	ns	*	4.258	3.196×10^{-3}
citrate	ns	ns	***	24.555	1.776×10^{-11}
**Lipid metabolism**					
LDL/VLDL	**	ns	**	14.361	3.336×10^{-8}
PUFA	ns	ns	*	7.588	3.753×10^{-5}
3-HB	ns	ns	ns	6.582	1.323×10^{-4}
glycerol	ns	ns	**	10.085	2.155×10^{-6}

Choline phosphorylation metabolism

GPC/PC	ns	ns	ns	6.818	9.787×10^{-5}

Note: Stars ***, **, * mean that the changes of relative metabolite levels in the mouse models are highly significant ($p<0.001$), very significant (p<0.01), significant (p<0.05) compared with those in the corresponding normal mice (aVMC *vs.* CON-w2, cVMC *vs.* CON-w6, DCM *vs.* CON-w24). Red and blue stars denote significant increase and significant decrease, respectively.

Table S3. Characteristic metabolites were determined by a combination of significant metabolites identified from the OPLS-Da (VIP > 1) and differential metabolites identified from the univariate analyses ($p < 0.05$).

Metabolite	aVMC *vs*. CON-2w	cVMC *vs*. CON-6w	DCM *vs*. CON-24w
acetate	↓	↑	↑
taurine	↓	↑	↑
LDL/VLDL	↑		↓
valine	↓	↑	
glycine	↓		
glucose	↓		↑
GPC/PC	↓		↓
lysine		↑	
glycerol		↑	
leucine		↑	
glutamine		↓	↑
PUFA		↑	↓
3HB			↑
succinate			↑
citrate			↑
creatine			↑

Note: The upward arrow and downward arrow denote that the difference between A and B is positive (A is increased compared to B) and negative (A is decreased compared to B), respectively.
